# Noninvasive optical monitoring of cerebral hemodynamics in a preclinical model of neonatal intraventricular hemorrhage

**DOI:** 10.3389/fped.2025.1512613

**Published:** 2025-03-10

**Authors:** Jyoti V. Jethe, YuBing Y. Shen, Edmund F. La Gamma, Govindaiah Vinukonda, Jonathan A. N. Fisher

**Affiliations:** ^1^ Department of Physiology, New York Medical College, Valhalla, NY, United States; ^2^ Department Pediatrics, Division of Newborn Medicine, New York Medical College, Valhalla, NY, United States; ^3^ Department of Biochemistry and Molecular Biology, New York Medical College, Valhalla, NY, United States; ^4^ Department of Cell Biology and Anatomy, New York Medical College, Valhalla, NY, United States

**Keywords:** intraventricular hemorrhage, cerebral blood flow, hemodynamics, diffuse correlation spectroscopy, microvascular flow, cranial ultrasound, germinal matrix, pediatrics

## Abstract

Intraventricular hemorrhage (IVH) is a common complication in premature infants and is associated with white matter injury and long-term neurodevelopmental disabilities. Standard diagnostic tools such as cranial ultrasound and MRI are widely used in both preclinical drug development and clinical practice to detect IVH. However, these methods are limited to endpoint assessments of blood accumulation and do not capture real-time changes in germinal matrix blood flow leading to IVH. This limitation could potentially result in missed opportunities to advance drug candidates that may have protective effects against IVH. In this pilot study, we aimed to develop a noninvasive optical approach using diffuse correlation spectroscopy (DCS) to monitor real-time hemodynamic changes associated with hemorrhagic events and pre-hemorrhagic blood flow in a preclinical rabbit model of IVH. DCS measurements were conducted during the experimental induction of IVH, and results were compared with ultrasound and histological analysis to validate findings. Significant changes in hemodynamics were detected in all animals subjected to IVH-inducing procedures, including those that did not show clear positive results on ultrasound 18 h later. The study revealed progressively elevated coefficients of variation in blood flow, largely driven by temporal fluctuations in the <0.25 Hz range. Our findings suggest that real-time optical monitoring with DCS can provide critical insights heralding pathological blood flow changes, offering a more sensitive and informative tool for evaluating potential therapeutics that may help avert the progression to IVH.

## Introduction

There are roughly 15 million premature births worldwide every year, accounting for nearly 11% of all births (1). Among the large number of other adverse health conditions, premature infants are susceptible to intraventricular hemorrhage (IVH) which is characterized by initial bleeding in the ganglionic eminence and subsequent accumulation of blood in the lateral ventricles. Reports indicate that roughly 45% of infants born before 32 weeks of gestation experience this condition in varying severity (2). Currently, no definitive preventive treatment is available. This burden is particularly acute in low-income countries due to limited access to advanced neonatal care (3). Premature infants are highly susceptible to the condition because of a host of developmental vulnerabilities in the germinal matrix that collectively render the developing microvasculature mechanically unstable and prone to rupture. This often arises from rapid intracranial pressure changes during the birthing process. The mechanical vulnerability largely reflects developmental idiosyncrasies of the blood brain barrier, such as a paucity of pericytes, low levels of fibronectin in the basal lamina, and reduced expression of glial fibrillary acidic protein (GFAP) in astrocyte end feet (4).

While most cases of IVH in premature infants are diagnosed within 7 days after birth, the greatest risk for bleeding occurs within the first 48 h of life (5). If increased risks of bleeding can be identified at an early stage, using preventive measures/timely intervention could help reduce the vulnerability of the germinal matrix vasculature. Early detection is crucial not only for minimizing further bleeding but also for preventing early injury and its long-term impact on neurological development, as well as for supporting the infant's physiological stability during the critical newborn period. Currently, clinical methods for detecting IVH include cranial ultrasound and magnetic resonance imaging (MRI) (6). Computed tomography (CT) is able to detect IVH, however it is not the initial methodology used for preterm newborns out of concern for exposure to radiation (7). Compared with other imaging modalities, ultrasound is by far the widely used clinical testing tool because it provides rapid, high-resolution images without the use of ionizing radiation. Other advantages include accessibility, portability, safety, and cost-effectiveness (8). Limitations of the technique, however, include operator-dependent variability in imaging quality and findings. Subtle irregularities in small fontanelles, for example, may be missed by a novice technician. Additionally, because ultrasound requires operation by a human technician, these diagnostics are generally performed at infrequent intervals, rarely at a rate greater than once daily (9); its utility is diagnosis after the event has occurred. In IVH, the main ultrasound imaging finding is hyper echogenicity due to fibrin formation (10), rather than identifying risks leading to active bleeding. Doppler ultrasonography can be used to detect blood flow (11) and has been used for continuous monitoring (12). However, the detected signals represent flow in large vessels rather than microvasculature, which is the predominant source of rupture in IVH (2). While advanced imaging approaches such as arterial spin labeling (ASL) MRI could potentially detect microvascular flow changes (13), the technique is not realistic for continual monitoring of premature infants and requires moving the patient to an MRI device, often necessitating additional sedation to capture quality images (14).

Diffuse optical technologies such as diffuse optical tomography (DOT) and near-infrared spectroscopy (NIRS) have found success in clinical adoption for real-time neuromonitoring of neonatal cerebral hemodynamics and tissue oxygenation (15). Pulse-DOT, for instance, can be used to measure cerebral hemodynamics in preterm infants including those who develop IVH (16). Diffuse correlation spectroscopy (DCS) is another emerging optical method that has demonstrated promise for monitoring tissue hemodynamics in real-time (17). DCS has been used effectively for non-invasive monitoring of cerebral hemodynamics and autoregulation in neurocritical care (18) and has also been applied to measurement of blood flow in infants with IVH (19). Whereas NIRS is typically used to probe cerebral metabolism based on tissue absorption properties, DCS utilizes temporal fluctuations of light scattered by moving red blood cells to obtain a relative blood flow index, which has units of cm^2^/s (20). When assessed alongside optical coherence tomography angiography *in vivo*, DCS was found to be preferentially sensitive to microvascular blood flow (21). Because the modalities can be integrated into the same probe, DCS and NIRS are complementary and have been implemented together with great success in clinical pediatric research. In particular, the combined measurement of cerebral blood flow and oxygenation in neonates is effective for detecting hypoxic ischemia (Gómez and Roblyer, 2024) (22) and monitoring brain health in cases of congenital heart disease (23, 24) and obstructive sleep apnea syndrome (25).

In this pilot study, we utilized a premature rabbit pup model to explore blood flow in the early phases of induced IVH. While rabbit and human premature neonates differ in aspects such as weight and gestation period, the rabbit model remains popular and translationally relevant for several reasons. Rabbits have a gyrencephalic brain structure with a greater proportion of white matter compared to rodents and the timing of perinatal brain white matter maturation is comparable to that in humans (26). Rabbits also have an abundant germinal matrix, a cortical blood supply from the carotid and vertebral arteries and, importantly, premature pups can survive IVH (27). Furthermore, premature rabbits are non-nocturnal and exhibit neuropathological consequences of preterm birth similar to those observed in human preterm infants (28). The glycerol induced IVH model of the rabbit pup brain closely mimics many of the clinical features of IVH such as hypomyelination, gliosis, pro-inflammatory cytokines, neurodegeneration, as well as cognitive delays seen in premature human neonates (29). Beyond assessing whether DCS could be utilized for detecting IVH, our secondary aim was to explore whether pre-hemorrhagic cerebral blood flow patterns in IVH-prone animals—glycerol injected animals, in our model—can be identified. Given that IVH initially entails dysfunction in the germinal matrix microvasculature, detecting the relevant hemodynamics requires imaging or neuromonitoring modalities that are sensitive to blood flow in capillaries in the ventricular wall.

## Methods

### Surgical procedures

**Figure 1:**
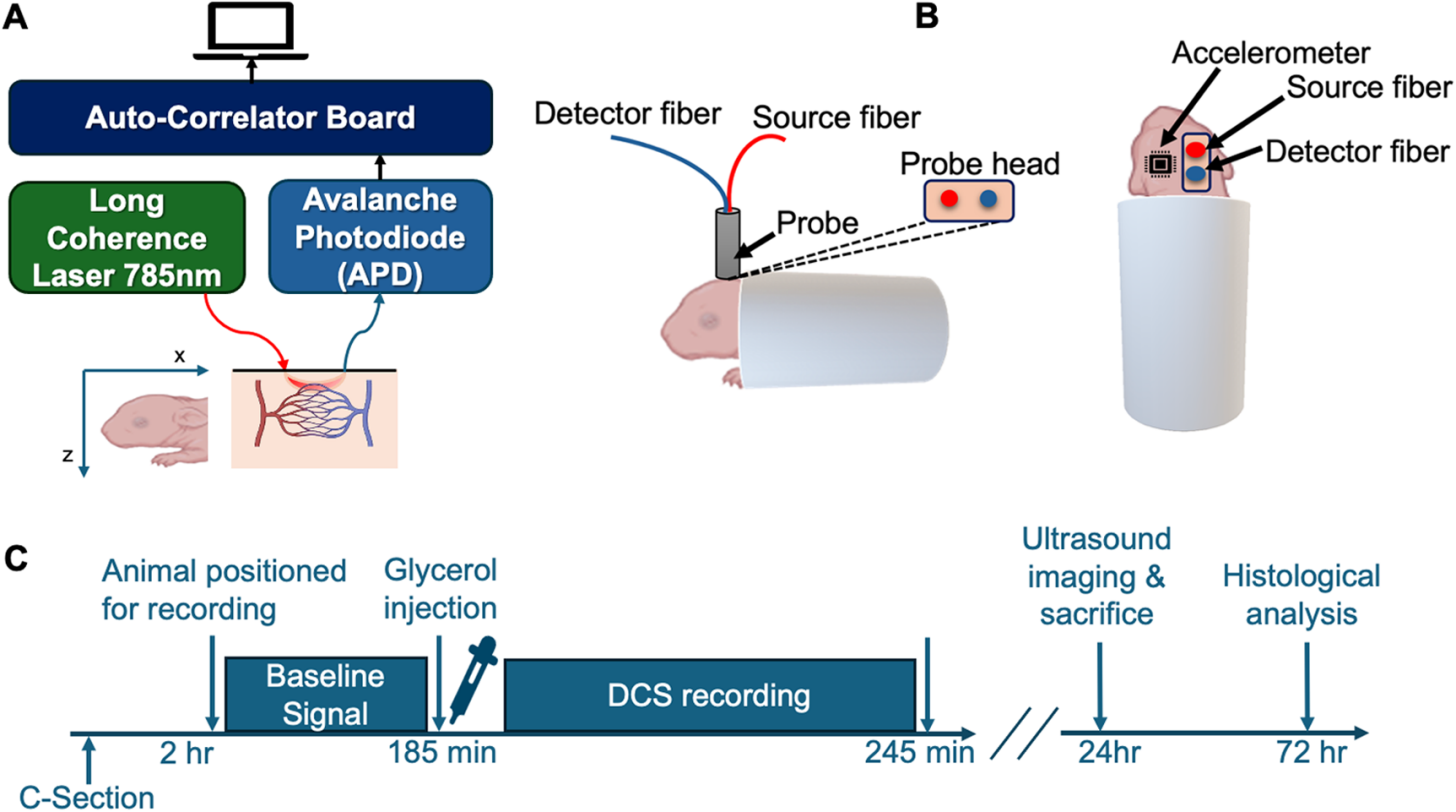
Experimental apparatus and protocol. **(A)** depicts a block diagram of the main components of the DCS system used in experiments. **(B)** illustrates the positioning of a premature rabbit infant and placement of the optical probe. Source-detector separation was 5 mm. **(C)** shows the experimental protocol and tissue processing timeline. Following acquisition of baseline flow signals for up to 25 min, glycerol (50%) was injected intraperitoneally. Optical signals were then continuously recorded for another 60 min. Cranial ultrasound was performed 24 h after birth, followed directly by euthanasia and perfusion for subsequent histological analysis.

All animal experiments were performed in accordance with the guidelines of the institutional Animal Care and Use Committee of New York Medical College. Timed pregnant New Zealand white rabbits whose pregnancy durations were carefully monitored were procured from Charles River Laboratories Inc. (Wilmington, MA). Premature pups were delivered via cesarean section at embryonic day 29 gestational age (rabbit full-term pregnancy is 32 days). Newborn premature pups, at 3–4 h of postnatal age, were secured in a custom padded capsule (Figure 1B) and treated with an intraperitoneal injection of 50% glycerol (6.5 g/kg, in physiological saline) which induces an osmotic IVH in approximately 70% of treated animals (29).

To determine the presence and severity of IVH, cranial ultrasound was performed ∼24 h after birth (Z. One SmartCart, ZONARE Medical System, Inc. CA, USA). The timing of screening was based on the fact that higher number of cases of IVH in premature infants occur within the first 72 h after birth (30, 31). In our prior work using this rabbit model, we have performed post-injection ultrasound imaging at 5 h, 24 h, 48 h, and 72 h and have found that, analogous to humans, ultrasound-detectable bleeding occurs within 5 h or else not at all (32–34). Diagnosis was based on the echogenicity within a region of interest (ROI) that spanned the ventricular area. For repeated ultrasound measurements, suspected germinal matrix rupture and bleeding was quantified based on the echogenicity ratio, which is the mean pixel value in a cranial ultrasound ROI divided by the corresponding value in either the same animal pre-injection or from a separate negative control animal.

### DCS system and data processing

The details of our DCS apparatus, depicted in Figure 1A, have been described previously (21). Briefly, 785-nm laser light from a long coherence length continuous-wave NIR laser (CrystaLaser, NV) was delivered to the animal's scalp via multimode optical fiber (200-μm core diameter, Fiberoptic Systems Inc, CA). Reemitted light was relayed from the scalp to a photon counting avalanche photodiode (APD) (SPCM-AQRH-12-FC, Excelitas, Quebec, Canada) via a single-mode fiber (5-μm core diameter, Fiberoptic Systems Inc, California). The output of the APD was directed to an autocorrelator signal processing board (Correlator.com), which computes an intensity autocorrelation function that is continuously sent via universal serial bus (USB) to a laptop PC (Dell Precision 7510) running LabVIEW software (National Instruments, Austin, TX) for further data analysis. Using custom code in MATLAB (ver. R2022b, Mathworks, Inc., Waltham, MA), a tissue cerebral blood flow index (CBFi) is obtained by fitting the measured intensity correlation function to a correlation diffusion equation (17) using an absorption coefficient µ_a_ = 0.1 cm^−1^ and reduced scattering coefficient of µ_s_’ = 8.0 cm^−1^. Source and detection fibers were integrated into a custom probe that was 3D printed using thermoplastic polyurethane (TPU) material (Shapeways, Inc.). The source-detector fiber separation was 5 mm. Spectral analysis was performed with EEGLAB (35) together with custom MATLAB code.

To monitor mechanical response and potential motion artifacts, the probe also integrated an accelerometer component (AST1001-BMA250, TinyCircuits) whose output was relayed to the same data acquisition laptop via USB. Individual CBFi measurements were acquired at 1 Hz and smoothed with 8s moving mean filter. The accelerometer acquires data in three orthogonal axes: x, y, and z. Small differences in the placement of the accelerometer can potentially lead to gain factors or offsets that are not physiologically meaningful, e.g., if an accelerometer is placed at a greater distance from points of flexion such as vertebrae and joints. Raw data from each axis was therefore min-max scaled, i.e., ${\overset{\cdot \cdot }{x}}_{norm}=\frac{\overset{\cdot \cdot }{x}\;-\;{\overset{\cdot \cdot }{x}}_{min}}{{\overset{\ldots }{x}}_{max}-{\overset{\cdot \cdot }{x}}_{min}}$. The accelerometer data's 3-dimentional magnitude was calculated by taking the square root of the sum of the squares of the acceleration values, i.e., $\sqrt{{\overset{\cdot \cdot }{x}}_{norm}^{2}+{\overset{\cdot \cdot }{y}}_{norm}^{2}+{\overset{\cdot \cdot }{z}}_{norm}^{2}}$. Blood flow fluctuations were quantified using the coefficient of variation (CV), defined as the ratio of the standard deviation (σ) to the mean (μ). Motion analysis was quantified based on the CV of the 3-dimensional magnitude of accelerometry data (CV_accel_). Analysis using CV enabled compensation for differences in offset, range, and noise level.

### Optical phantom experiments

To verify that optically-derived CBFi values and mechanical measurements were independent, we performed optical “phantom” experiments where phenomenological “flow” was controlled using optically detected Brownian motion in aqueous intralipid emulsions of varying viscosity (36). A 1-gallon black polypropylene bucket was filled with a solution of water, glycerol, and intralipid. In this setup, a probe was inserted into the bucket, with an accelerometer attached to the probe allowing simultaneous acquisition of both flow signals and accelerometer data. High and low flow rates were simulated with glycerol concentrations of 2.26 M and 4.52 M, respectively.

### Experimental protocol

After animals were positioned, the 3D-printed probe, which was stabilized on a mechanical isolation table with optomechanic posts (Thorlabs, NJ), was carefully lowered onto the animal's scalp above the region of the forebrain. This scalp position corresponds to a region atop lateral ventricles on the mid-sagittal line and Bregma (29). A total of six premature rabbit infants were selected as subjects for the experiment. The study involved 5 animals injected with glycerol and a sham animal that was positioned and measured identically but did not receive glycerol injection. As depicted in the timeline in Figure 1D, following positioning in the measurement apparatus, hemodynamics were monitored for a period of 5 min, after which non-sham animals were injected with glycerol. Following the injection, optical measurements resumed for at least one hour. This interval of time was selected to minimize the animals’ discomfort due to restraint. Animals were then returned to a neonatal incubator set at 27°C. A cranial ultrasound was performed on the animals 24 h after the DCS recordings. Although neonatal rabbit pups are born with closed eyes, ears and an incompletely developed auditory system, our optical measurements were made in an acoustically-attenuated room to minimize ambient acoustic noise.

### Histological analysis

To measure the ventricular cross-sectional area, coronal brain sections were prepared using hematoxylin-eosin (H&E) staining (29). First, the rabbit pups were perfused with 1 × phosphate-buffered saline (PBS) buffer. Brains were dissected out and postfixed in 4% paraformaldehyde for 24 h at 4℃, after which they were dehydrated with 15% sucrose for 12 h and then with 30% sucrose for 12 h. Brains were then embedded in OCT compound (Tissue-Tek, Sakura Finetechnical Co., Tokyo, Japan) and sectioned into 18-µm thick coronal sections on a cryostat (CM1850, Leica Biosystems) (37). The H&E staining procedure was performed in accordance with our previously published methods (29). Briefly, sections were air-dried at room temperature (RT) for 20–30 min, followed by rinsing in PBS for 5 min (2 washes). Sections were fixed with acetic acid for 1 min, rinsed with water, and stained with hematoxylin for 1 min. After an additional water rinse, sections were treated with ammonia water and rinsed again. They were then exposed to 95% ethanol for 15s, stained with eosin for 30s, and sequentially dehydrated in 95% ethanol (2 washes), followed by 100% ethanol, and cleared with three immersions in xylene. After staining the images were created using a microscope (Keyence, BZ-X810).

## Results

**Figure 2:**
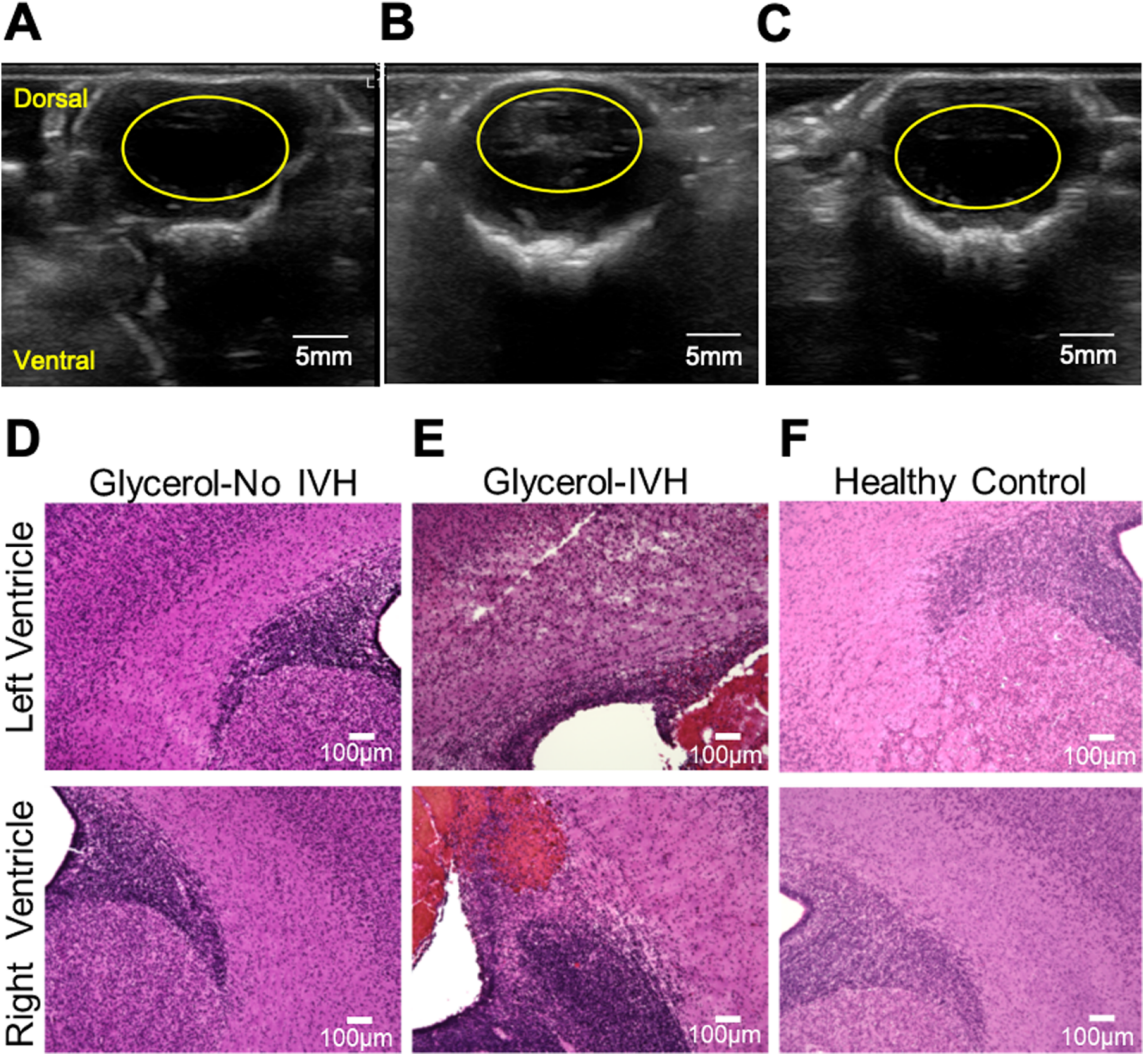
Validation of IVH by means of ultrasound and tissue histology. **(A-C)** show representative ultrasound imaging results obtained 24 h following experiments. The images correspond to representative IVH-, IVH+, and sham animals. In these cross-sectional images, the yellow ovals indicate the ventricular region. An example of increased echogenicity in the lateral ventricles can be seen within the yellow oval in **(B) (D-F)** show representative images of 18-μm-thick H&E stained coronal brain slices from representative IVH-, IVH+, and sham animals, in sequence. Extravasated erythrocytes within the ventricular lumen can be seen in **(E).**

The experimental protocol and DCS apparatus for monitoring hemodynamics are shown in Figure 1. Cranial ultrasound imaging performed 24 h after birth validated IVH in 3 out of 5 injected animals based on increased echogenicity in the lateral ventricles (Figures 2A–C). H&E staining of coronal brain sections from animals with IVH sacrificed directly following ultrasound imaging revealed extravasated erythrocytes within the ventricular lumen (Figure 2D–F). In non-injected control animals, no increased echogenicity was observed in ultrasound 24 h after injection (Figure 2C) and the germinal matrix did not appear compromised (Figure 2F).

**Figure 3:**
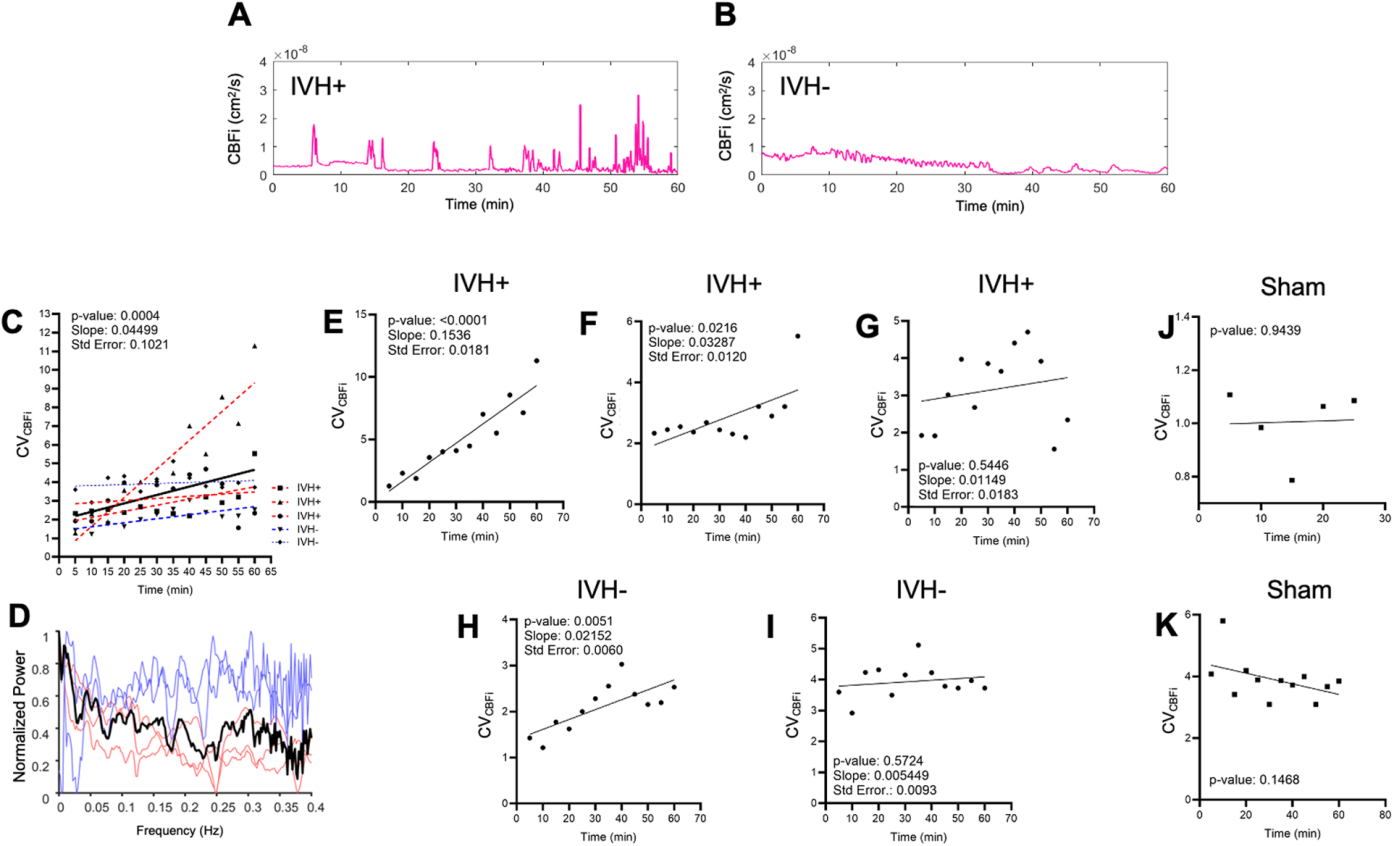
Optically recorded cerebral blood flow index (CBFi) dynamics following glycerol injection. **(A,B)** show cerebral blood flow recorded continuously for 60 min immediately after intraperitoneal (IP) injection of 50% glycerol. The animal in **(A)** was classified as IVH + based on cranial ultrasound and histology 24 h post-injection, whereas the animal in **(B)** was classified as IVH-. **(C)** presents a linear fit of CV_CBFi_ data from all injected animals (*N* = 5). Each data point represents CV_CBFi_ averaged over a 5-minute window in one animal. Data points from individual animals are distinguished by marker shape and color (red: IVH+, blue: IVH-). The linear fits for individual animals are also shown superimposed as thinner dashed lines with the same red/blue color scheme. Panel **(D)** presents normalized frequency spectra (0.002–0.4 Hz) for the five injected animals. Light blue and red traces are from individual IVH- and IVH + animals, respectively, and the black trace shows the normalized average of these individual spectra. **(H,I)** depict coefficient of variation analysis of CBFi (CV_CBFi_) for all injected and sham animals. CV_CBFi_ measurements derived from individual IVH + animals are shown in **(E–G)** and from IVH- animals in **(H,I)**. CV_CBFi_ measurements obtained from two uninjected sham animals are shown in **(J,K)**. Standard error (Std. Error) represents the uncertainty in the slope fit.

Following glycerol injection, optically recorded hemodynamics waveforms were complex and varied among animals, as can be seen in the representative traces in Figures 3A,B. Despite animal-to-animal variability in the raw blood flow signals, however, the coefficient of variation of cerebral blood flow (CV_CBFi_) increased following injection when summed over all animals. Figure 3C depicts the CV_CBFi_ results of all animals, each datapoint averaged in consecutive windows of 5 min, together with the results of linear regression that reveal a significant positive slope (*P* = 0.0004). Figure 3D presents a normalized power spectrum for CBFi, displaying both individual experimental results and the average spectrum. The spectra for individual animals represent analysis over the full 60–90 min DCS recordings. Although there is considerable variability between animals, the averaged spectrum depicts broad, albeit noisy, peaks below 0.25 Hz. Frequencies above ∼0.3 Hz exhibit patterns consistent with random fluctuations. Figures 3H,I show the results of individual animals, including IVH + (Figures 3E–G) and IVH- (Figures 3H,I) animals. Two of the three IVH + animals (Figures 3E,F) and one of two IVH- animals (Figure 3H) yielded statistically significant positive slopes following injection. As can be seen in Figure 3D, IVH + animals exhibited a greater mean rate of increase over the course of 60 min following injection compared with IVH- animals, however the sample size was too small to test for significance. Nonetheless, linear regression yielded a positive CV_CBFi_ slope for both IVH- animals, only one of which was significant.

To explore whether apparent hemodynamic signals could have been a result of motion artifacts, we performed the same CV analysis on the 3-dimensional magnitude of accelerometry data (CV_accel_) and found no overall significant change over the course of the experiment for all animals (Supplementary Figure S1A). Similarly, we found no significant dependence between CV_accel_ and CV_CBFi_ in the two sham animals (Supplementary Figure S1B). To verify that flow and accelerometry measurements were independent, we performed phantom measurements wherein flow was measured when the probe was immersed in highly scattering lipid emulsions of varying Newtonian viscosity. As shown in the scatterplot depicted in Supplementary Figure S1C, increasing viscosity (thus reducing the phenomenological flow index) did not alter the relationship between CV_accel_ and CV_CBFi_. Sample raw accelerometry data as well as simultaneously recorded CBFi are shown in Supplementary Figures S1D,E, respectively.

**Figure 4:**
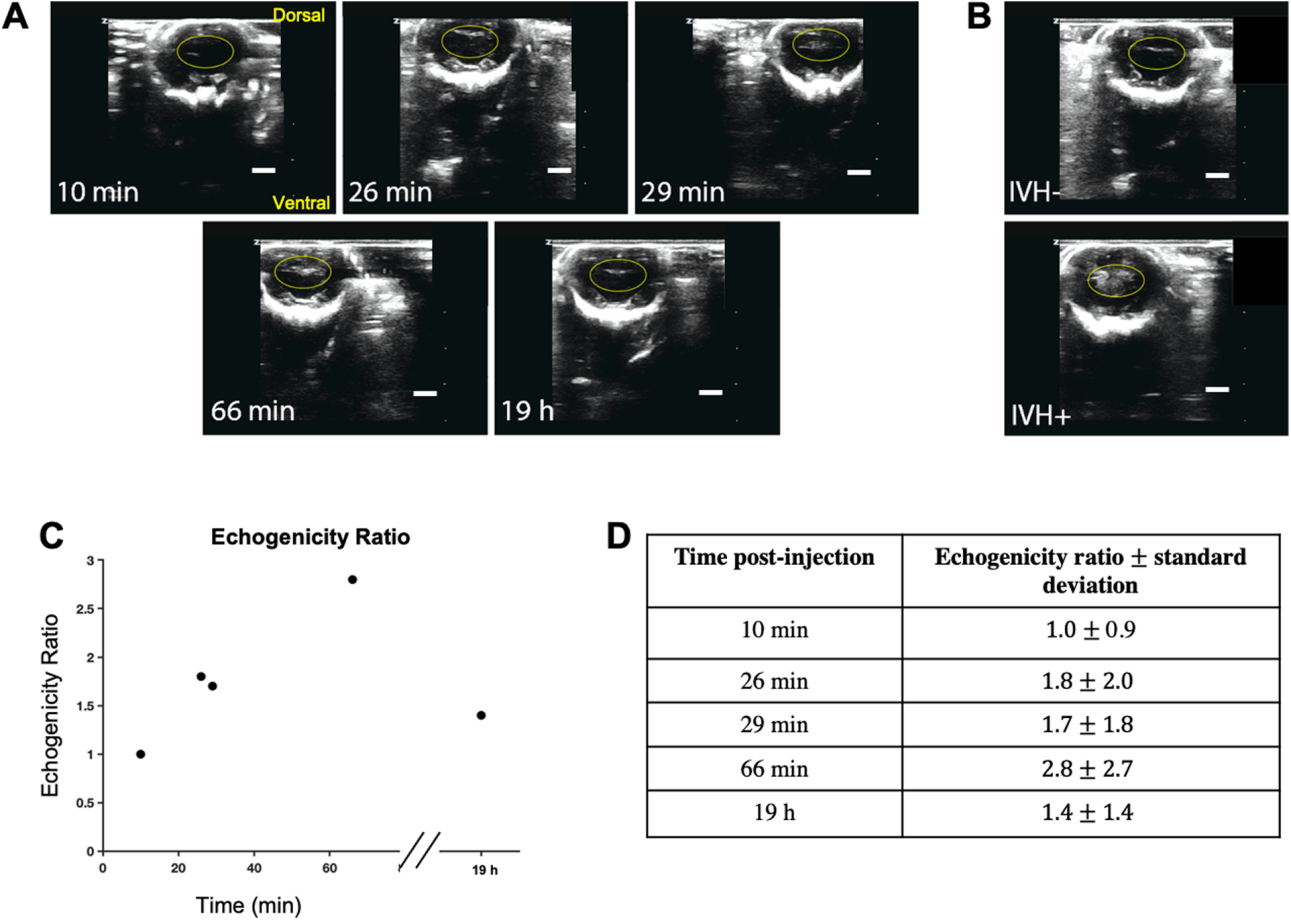
Repeated cranial ultrasound provides evidence for acute germinal matrix bleeding in glycerol-injected animal. **(A)** shows repeated ultrasound measurements at time points indicated in the lower left corner of each image; the values refer to the elapsed time following injection. Monitoring the same ROI, it is apparent that echogenicity increases upward of 1 h post injection, yet by 19 h has returned to near baseline values. **(B)** shows representative cranial ultrasound coronal views of animals one day after glycerol injection; the top image is from an IVH- rabbit pup, and the bottom is IVH+. Increased echogenicity can be observed within the ventricular region highlighted with a yellow oval. **(C)** quantifies these trends by showing the echogenicity ratio, which was calculated as the ratio of averaged intensity within the ROI (shown as the ovals) for each timepoint divided by the average intensity of the initial ROI. **(D)** tabulates the echogenicity values for the timepoints in the plot in **(C)** Scale bar: 5 mm.

With our current DCS detector probe form factor, it was not practical to perform simultaneous, continuous cranial ultrasound measurements. Additionally, cranial ultrasound involves considerable manipulation of the animal to obtain a clear cross-sectional image of the ventricles. Nonetheless, in an attempt to correlate our hemodynamic measurements with a gold standard diagnostic modality, we performed repeated cranial ultrasound imaging in a separate animal over a time period that matched our DCS experiments (Figure 4). The animal, which was not measured using DCS, was injected with glycerol and ultimately diagnosed as IVH- 19 h later based on low levels of echogenicity in the lateral ventricles (Figure 4A). Despite this terminal diagnosis, we recorded an increase in the echogenicity that emerged as early as 26 min and which continued to increase upwards of 1 h. For reference, Figure 4B shows terminal end point ultrasound measurements in a separate IVH + and IVH- mouse. We quantified the acute progression in terms of an echogenicity ratio, which was calculated as the ratio of the mean pixel value within the yellow oval ROI divided by the corresponding mean ROI value observed at the 10 min timepoint. As shown in Figure 4C, by 66 min the ratio reached 2.8 ± 2.7 (mean ± standard deviation of pixel values) before falling to 1.4 ± 1.4 19 h later. For comparison, the echogenicity ratio of the IVH + animal (Figure 4B bottom) 18 h post-injection relative to the uninjected control animal (Figure 4B top) was 2.9 ± 3.4.

## Discussion

Near infrared diffuse optical neuromonitoring has been applied to rabbit models for detecting induced hypoxia (38, 39), bacterial infection (40), and seizures (41). Our work extends optical monitoring in rabbits to include cerebral blood flow rather than oxygenation and metabolism metrics. Our main finding of heightened variability in CBFi following DCS likely reflects impaired autoregulation of cerebral blood flow owing to the significant physiological insult caused by the glycerol injection even prior to the occurrence of IVH. In healthy newborns, cerebral autoregulation ensures that cerebral blood flow is not heavily influenced by systemic blood pressure, albeit over a much narrower range of blood pressure compared to adult animals. Injecting glycerol intraperitoneally results in fluid shifts and an elevation of serum osmolarity that induces selective rupture of vasculature in the fragile immature microvascular bed of the germinal matrix (42). The fluctuations that we observed were largely in the frequency range below 0.25 Hz (Figure 3D). This is consistent with dynamics that have been observed in a rabbit model of ischemic stroke (43). Furthermore, increases in CV_CBFi_ variability began roughly 5 min post injection, which closely matches the onset of rapid increase in jugular venous pressure following IP injections of 50% glycerol in rats (44). Given the vulnerability of microvasculature in the germinal matrix, we hypothesize that the fluctuations in CV_CBFi_ reflect flow dynamics in that vascular population. The observation that IVH did not occur in all glycerol-injected animals aligns with prior studies using this rabbit model (29). While the CV_CBFi_ slope trend in IVH- animals did not achieve statistical significance within our sample size when analyzed separately from IVH + animals, the difference in CV_CBFi_ trajectories may represent a reduced level of cerebral autoregulation impairment or else an early recovery from the physiological insult.

The repeated cranial ultrasound measurements in a separate IVH- animal offer evidence from a gold standard diagnostic that IVH- animals can, in fact, exhibit significant bleeding within the first ∼1 h of glycerol injection (Figure 4). While it was not practical to perform simultaneous cranial ultrasound in parallel with our DCS measurements, our results demonstrate that this method could be used in future experiments to validate the ability to distinguish IVH- from sham animals based on CBFi. Standard clinical cranial ultrasound is not particularly sensitive to microvascular flow, however. For validating the assertion that the signals recorded by DCS truly reflect microvascular flow in the ventricular wall, some possible approaches include the use of ASL (45), optical coherence tomography angiography (OCT-A) in conjunction with cortical windows (46), or super-resolution ultrasound imaging, which can map flow in vessels as small as ∼10 μm diameter (47, 48).

IVH studies in humans, while more directly clinically relevant than preclinical models, are challenging due to the unpredictable timing of IVH onset. Prior work applying DCS to IVH in preterm infants, for example, utilized a case-control study design to retrospectively explore CBFi dynamics that were potentially predictive of IVH (19). While elevated CV_CBFi_ was observed in infants who later developed IVH, the correlation did not reach statistical significance in those prior studies. This is likely because measurements were not obtained imminently before IVH, which is spontaneous. Our preclinical model, however, facilitates a more direct path to identifying real-time quantitative cerebral blood flow patterns that can predict IVH because it is experimentally induced.

The overall significance of our proof-of-concept experiments is the promise that hemodynamics associated with the onset of IVH can be monitored in unanesthetized small animals using an established preclinical model IVH that mimics the human condition. Limitations of this study include the fact that we were unable to fully distinguish the hemodynamic trends of IVH + vs. IVH- animals, presumably due to the modest sample size. Additionally, although the repeated cranial ultrasound measurements provide evidence that acute ventricular bleeding can occur in IVH- animals, future experiments must include simultaneous DCS and ultrasound to confirm that hemodynamic changes such as the ones depicted in Figure 3 correlate with increased echogenicity. Beyond random physiological variability between animals, the nonlinear dynamic nature of hemorrhagic events significantly complicates both our experimental design and statistical interpretation because the system is at a mathematical critical transition (49). In addition to increasing sample size, future studies can therefore use mixture modeling approaches to help identify and characterize the hemodynamic trajectories of IVH + vs. IVH- subgroups (50).

In terms of translatability of this preclinical work, while our current probe design is not optimal for applying to human neonates, a number of studies have developed DCS probes that are fit-for-purpose (22–25). We therefore do not anticipate ergonomic factors as a barrier. Prior to clinical translation, however, a thorough characterization of differences in skin optical properties between the rabbit model and human neonates is needed. While a comprehensive study is currently lacking, the range of reported values for human skin thickness, at least, is within the range of rabbits’ (51, 52). Differences in skin pigmentation could also impact the inferred CBFi signals. However, recent work has found that differences in skin pigmentation, quantified by the individual typology angle (ITA), have a minimal impact on DCS interpretation (53).

Beyond simply detecting the presence of IVH, identifying clinically relevant, stereotyped fluctuations of microvascular CBF that are predictive of IVH could have a significant impact on clinical practice. Historically, before 2007, IVH was most commonly identified within 6 h of birth (35%) yet when using more contemporary perinatal-neonatal management strategies, after 2007, the rate fell to just 9% (31) where nearly all remaining events arose in the first week reflecting risks associated with postnatal interventions or progression of an antecedent hypoxic injury. This delay affords the neonatal clinician a window of opportunity to use DCS to identify suspicious patterns in CBFi before the actual IVH occurs. Early identification of impending IVH in at-risk neonates (e.g., <28 weeks gestational age) may offer sufficient time to make adjustments in clinical management that can mitigate the problem (e.g., minimizing barotrauma with the ventilator or altering CNS blood flow due to extremes of pH). Additionally, predictive hemodynamic signals could serve as functional endpoints for developing new therapeutic interventions. Real-time measurements would provide an alternative to the current gold standard methods such as ultrasound or histology which yield delayed, binary IVH diagnoses and may lead to “no-go” decisions for otherwise promising drug design approaches. We speculate that this enabling report will incentivize clinicians to perform clinical trials in human newborns and may even help identify opportunities for novel therapies including use of stem cells (29).

## Data Availability

The raw data supporting the conclusions of this article will be made available by the authors, without undue reservation.
