# Supplementary material for: Noninvasive optical monitoring of cerebral hemodynamics in a preclinical model of neonatal intraventricular hemorrhage
Source: Front Pediatr. 2025 Mar 10;13:1512613. doi: 10.3389/fped.2025.1512613 (PMC11930821; doi:10.3389/fped.2025.1512613)
Supplement: Supplementary file 1 [file Datasheet1.pdf]

Supplementary Materials

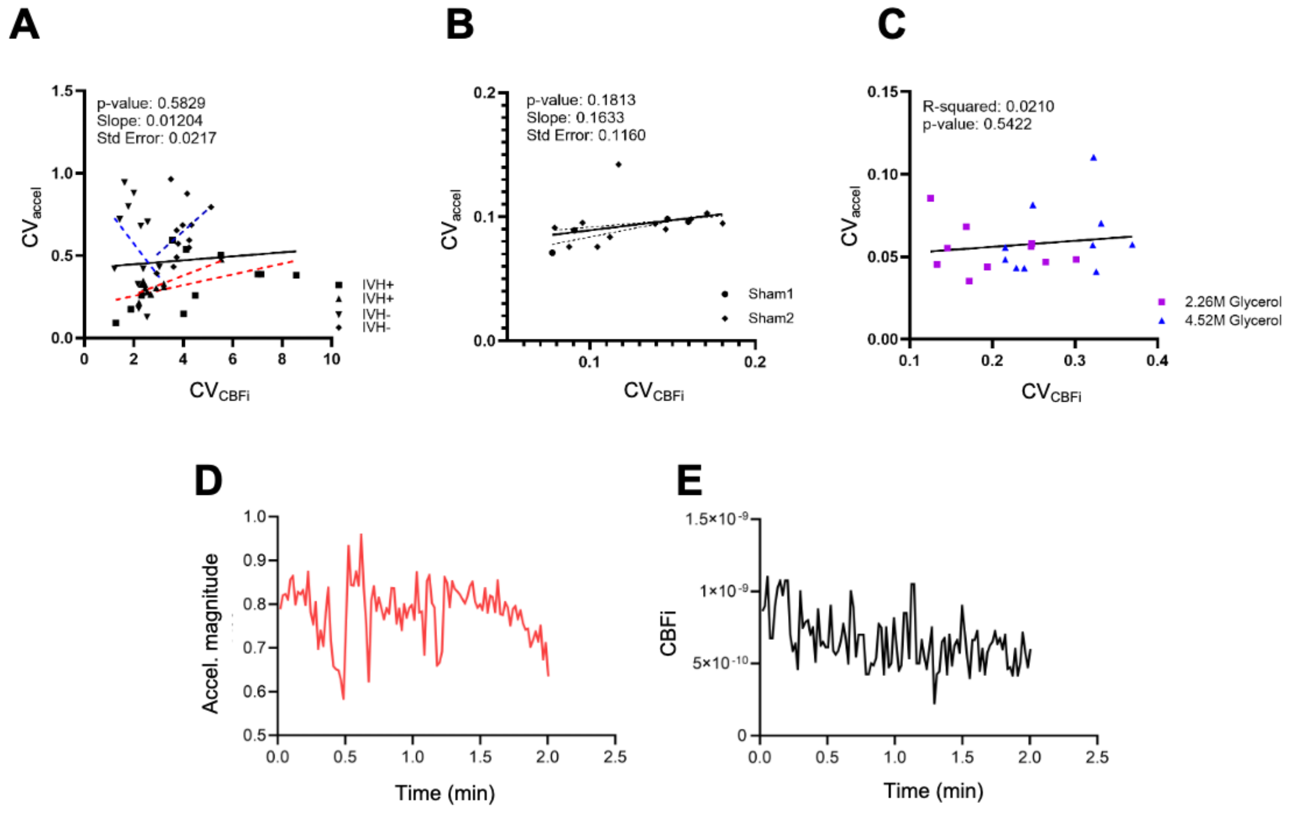

Figure S1: Physiological insult induces no significant motor or mechanical artifacts. **A** shows a plot of CV_{accel} vs. CV_{CBFi} for all experiments where accelerometry was possible (N = 4). Each data point represents CV_{accel} averaged over a 5-min window. Measurements from different individual animals are distinguished based on marker shape and color (red: IVH+, blue: IVH-) and the superimposed colored lines show the result of linear regression for individual animals. **B** shows a linear fit to a plot of CV_{accel} vs. CV_{CBFi} for uninjected sham animals (N = 2). **C** depicts the results of phantom measurements involving concurrent monitoring of 3D acceleration and DCS measurements of flow. The optical and mechanical sensors were bound and immersed in a highly-scattering intralipid solution at two viscosities. Each data point represents CV_{accel} averaged over a 2 min window of acceleration paired with a CV_{CBFi} within the same time window. All datapoints of one color are derived from a single experiment where data was acquired for 5 min. Std. Error corresponds to the standard error of the slope fit. Panel **D** shows a sample trace of raw accelerometer 3-axis magnitude, min-max normalized and panel **E** shows the corresponding CBFi for the recorded simultaneously via DCS.
